# Dopamine Transporter Imaging, Current Status of a Potential Biomarker: A Comprehensive Review

**DOI:** 10.3390/ijms222011234

**Published:** 2021-10-18

**Authors:** Giovanni Palermo, Sara Giannoni, Gabriele Bellini, Gabriele Siciliano, Roberto Ceravolo

**Affiliations:** 1 Unit of Neurology, Department of Clinical and Experimental Medicine, University of Pisa, 56126 Pisa, Italy; giovanni.palermo@med.unipi.it (G.P.); giannonisara@gmail.com (S.G.); g.bellini3@studenti.unipi.it (G.B.); gabriele.siciliano@unipi.it (G.S.); 2 Unit of Neurology, San Giuseppe Hospital, 50053 Empoli, Italy; 3 Center for Neurodegenerative Diseases, Unit of Neurology, Parkinson’s Disease and Movement Disorders, Department of Clinical and Experimental Medicine, University of Pisa, 56126 Pisa, Italy

**Keywords:** biomarkers, dopamine transporter, SPECT, nigrostriatal pathway

## Abstract

A major goal of current clinical research in Parkinson’s disease (PD) is the validation and standardization of biomarkers enabling early diagnosis, predicting outcomes, understanding PD pathophysiology, and demonstrating target engagement in clinical trials. Molecular imaging with specific dopamine-related tracers offers a practical indirect imaging biomarker of PD, serving as a powerful tool to assess the status of presynaptic nigrostriatal terminals. In this review we provide an update on the dopamine transporter (DAT) imaging in PD and translate recent findings to potentially valuable clinical practice applications. The role of DAT imaging as diagnostic, preclinical and predictive biomarker is discussed, especially in view of recent evidence questioning the incontrovertible correlation between striatal DAT binding and nigral cell or axon counts.

## 1. Introduction

Parkinson’s disease (PD) is a chronic, progressive neurodegenerative disorder characterized primarily by motor symptoms which are attributed mostly to dopaminergic cell loss. However, this is just the “tip of the iceberg” and a range of non-motor disturbances related to a combination of dopaminergic and non-dopaminergic pathways indicates the multi-focal and widespread driven pathology of PD [1,2]. As the diagnosis of PD remains clinical, this heterogeneity can pose diagnostic difficulties in line with clinico-pathologic series reporting suboptimal diagnostic accuracy in the early stages, even at specialized centres [3,4]. A correct diagnosis, however, is important for patient counselling and clinical research purposes. Similarly, rate of disease progression is also heterogeneous so that the identification of markers of progression would be of utmost importance to inform prognosis and timely offer the best therapeutic options for the advanced phases. Dopaminergic medications remain the major therapeutic approach for controlling clinical symptoms of PD and improving the quality of life of patients for many years, but over the years they lose efficacy and are associated with significant complications such as the “wearing off” effect and dyskinesias. There are currently no disease-modifying therapies for PD, heightening the challenges for the public health system posed by the epidemiology of PD, with numbers projected to double in the next few decades [5]. In this context, the validation and standardization of pathophysiological biomarkers which aid clinicians for early detection, prognosis, tracking disease progression and prediction of treatment response, represents an unmet and urgent need to be addressed for improved clinical assessment and management of PD patients.

Positron emission tomography (PET) and single photon emission tomography (SPECT) imaging with specific dopamine-related tracers offer a practical indirect imaging biomarker of PD, serving as a powerful tool to assess the status of presynaptic nigrostriatal terminals. More frequently, the nigrostriatal dopaminergic function is assessed in vivo with several [^123^I] and ^99m^Tc-labeled SPECT agents that measure the dopamine transporter (DAT) availability. In particular, the radiotracer Ioflupane or [^123^I]-FP-CIT has been approved by both FDA (DaTscan™) and EMA (DaTSCAN) for clinical use in suspected parkinsonian syndromes, becoming part of the diagnostic guidelines of α-synucleinopathies (PD, multiple system atrophy and dementia with Lewy bodies) [6,7]. In this review, we will discuss the role of DAT imaging as a biomarker in PD for diagnosing, monitoring progression, determining disease severity and predicting clinical outcomes over the course of PD, with particular emphasis on recent updates about the relationship between DAT availability and dopaminergic nigrostriatal neurons.

## 2. Targets of Dopaminergic Imaging

In clinical setting there are an increasing number of molecular targets for dopaminergic system imaging. The analysis can be performed at a presynaptic level, where we can evaluate the L-aromatic amino acid decarboxylase (AADC), the vesicular monoamine transporter 2 (VMAT2) and the DAT density, or at postsynaptic level estimating dopamine receptors expression [8].

**Figure 1:**
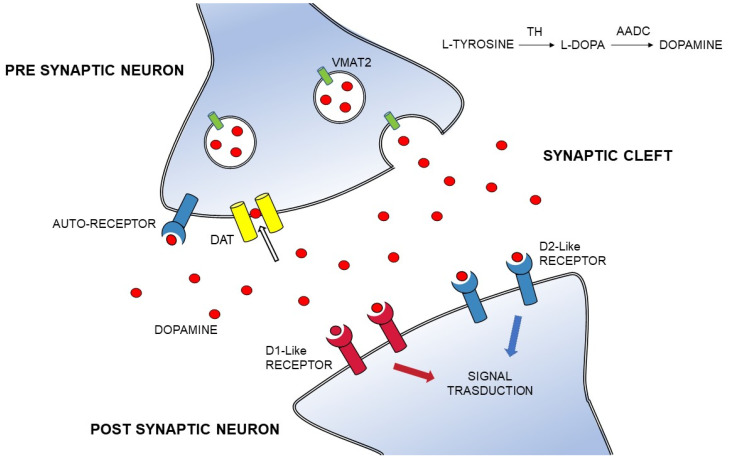
The dopaminergic synapse. DA is derived from the L-tyrosine metabolism to L-dopa mediated by TH and AADS; the DA transport into synaptic vesicles is operated by VMAT-2; the DA synthesis is regulated via presynaptic auto-receptors (in blue); the presynaptic neuron releases DA (red circles); DA binds two main types of receptors (D1 family formed by D1-D5 receptors and D2 family formed by D2-D3-D4 receptors) on the post-synaptic neuron; presynaptic DAT (in yellow) regulates the DA reuptake from the extracellular fluid. Abbreviations: DA, Dopamine; AADC, aromatic L-amino acid decarboxylase; DAT, dopamine transporter; TH, tyrosine hydroxylase; VMAT-2, vesicular monoamine transporter 2.

DAT is a transmembrane sodium chloride dependent protein selectively expressed in dopaminergic cells. DAT has a key role in the dopamine (DA) reuptake from the synaptic cleft, and it is critical in the spatial and temporal buffering of released DA levels [9] (Figure 1). Indeed, DAT modulates quantal DA release at endplates regulating also DA storage in synaptic vesicles [10]. DAT expression on presynaptic terminals reflects striatal dopaminergic innervation, implicating a direct relationship between its reduction and nigral cell loss. Imaging of DAT in PD confirms a significant reduction of striatal uptake, with a direct correlation between decreased DAT in the putamen and PD symptoms [11]. [^123^I]-FP-CIT is the most used radioligand for DAT because of its high affinity, specificity and fast pharmacokinetic profile [12]. Other DAT ligands frequently used to evaluate in vivo the dopaminergic system are [^99m^Tc]TRODAT, [^123^I]-β-CIT, ^18^F-FP-CIT and [^123^I]IPT, but their kinetic properties are slightly different [13,14,15]. Specifically, DAT-SPECT imaging is interpreted qualitatively based on the visual interpretation, but semi-quantitative assessment using striatal region of interests is often commonly performed in the routine clinical setting. Visual assessment is usually sufficient for clinical evaluation, but quantitative analyses of images may provide more objective measurement of striatal binding ratios [16,17]. However, both methods demonstrated an almost perfect agreement rate even though with some challenges and potential inaccurate results due to human error [18]. Besides its high affinity for DAT, it is important to remember that [^123^I]-FP-CIT also has affinity for other monoamine transporters, such as the serotonin transporter (SERT) and the norepinephrine transporter (NET). Notably, extrastriatal binding of [^123^I]-FP-CIT seems to derive from serotonergic transporters density, which is found mainly in the midbrain and diencephalon, suggesting DAT-SPECT as a proxy for the integrity the extrastriatal serotonergic system [19].

## 3. Confounding Factors on the Interpretation of DAT

Aging and gender can both influence PD incidence, symptoms and treatment response but few studies have examined sex differences and aging effects on DAT in PD patients [20,21].

Progressive decline of DAT with aging is extensively reported in healthy subjects, with an average decline between 4% to 8% per decade [22,23]. Interestingly, experimental and functional data have led to the suggestion that DAT could be down-regulated in intact nerve terminals in response to age-related changes in the basal ganglia, suggesting a possible compensatory phenomenon which contributes to offset a natural decline in motor function observed in older individuals [24,25,26]. In PD there are mixed reports about the influence of aging on DAT, partly because of difficulty standardizing the severity of disease [27,28]. Lee and colleagues demonstrated a smaller-than-normal age effect on DAT ligand binding in the putamen, but not in the caudate of de novo PD patients [29]. The greater disease-related degeneration of putamen in early PD may mask age-related correlations, advocating for a possible floor effect in putaminal DAT binding in PD patients. However, this imbalanced age-decline across striatal subregions could be compatible with compensatory responses in the putamen to further DA losses, superimposed upon even age-related changes [29]. Similarly, in another study on a large sample of PD patients, a significant relationship between the age and DAT uptake was restricted to the caudate nuclei, without differences in the rate of decline between PD patients and controls [20]. In addition the authors described a concurrent decline of [^123^I]-FP-CIT binding in several extrastriatal regions, except for the SERT-rich midbrain [20].

Sex differences in the dopaminergic system have been similarly reported, with most evidence of higher DAT availability in women than men which could be related to higher dopamine transmission/turnover or greater volume of the striatum for the beneficial influence of estrogens [23,30,31,32]. Accordingly, female patients would exhibit a more rapid age-related decline in DAT activity than male patients as a consequence of lowering levels of estrogen with aging [33]. However, other studies reported equivalent dopaminergic binding between sexes [34,35,36] or a gender effect limited to young-to-middle aged healthy subjects, with no significant differences in the elderly age group [37].

Functional molecular imaging studies in PD patients reported a similar trend with higher DAT binding in women than men at both symptoms onset and during the disease progression [38]. This gender effect seems to be more prominent in the caudate than in the putamen [20]. 

The influence of dopaminergic drugs on DAT binding has been explored in neuroprotection trials of early PD patients which used striatal DAT-SPECT as an index of disease progression [39,40]. Imaging findings from the CALM-PD-CIT or REAL-PET studies raised the possibility that both dopamine agonists (pramipexole and ropinirole) could exert neuroprotective effects or that levodopa could be toxic for nigral cells in PD [41,42]. Notwithstanding, no corresponding clinical benefit in favor of the dopamine agonists was observed and it is difficult to separate their direct effects on tracers from their effects on disease progression [43]. Actually, another interpretation of these data is that imaging measures may simply reflect different effects of dopamine agonists and levodopa on the regulation of DAT expression [44,45]. Interestingly, a possible mild upregulation of DAT after chronic treatment with rotigotine has been interpreted as the consequence of a different occupancy of D1 versus D2 postsynaptic receptors when compared with the other dopamine agonists [46]. Overall, there is no consensus on the direction of the change in striatal DAT levels caused by dopaminergic drugs and, in the majority of cases, no change has been found [47]. All drugs with high affinity for the DAT and SERT (such as selective serotonin reuptake inhibitors), may result in a higher striatal binding ratio, whereas sertraline may block DAT leading to decreased striatal tracer uptake [48,49]. However, their effects appear to be only around 10%, arguing that it could be therefore not necessary to withdraw these medications for routine clinical studies. Accordingly, patients are currently submitted to DAT-SPECT study without withdrawing dopaminergic medications [50]. Conversely, all kinds of drugs which may lead to a misleading scan should be excluded in scientific studies, when even small differences may be relevant [51]. Among the central nervous system stimulants, cocaine, amphetamines, methylphenidate and modafinil are high-affinity DAT blockers [52]. Other medication and drug abuse that may significantly influence the interpretation of DAT-SPECT by reducing DAT levels include ephedrine, some antidepressants (e.g., bupropion), anticholinergic drugs (e.g., benztropine), opioids (e.g., fentanyl) and anesthetics (e.g., ketamine) [50]. In addition, it has been suggested that drugs increasing plasma levels of the adrenergic agonists may be able to affect striatal binding of DAT [53]. A recent study showed that zonisamide treatment, which has been authorized as add-on treatment for PD patients in Japan, can delay the reduction of striatal DAT levels in relatively early-stage PD patients [54]. Instead, it is unlikely that other common medications, including tricyclic antidepressants, amantadine, neuroleptics and cholinesterase inhibitors, will influence DAT imaging significantly. Finally, it should be noted that other habits, such as tobacco and cannabis addictions, may interfere with dopaminergic brain circuits, decreasing DAT availability [55,56], but large differences between smokers compared to ex-smokers and healthy volunteers have been excluded [57]. 

Finally, although some studies suggest an association between polymorphisms in the gene coding for DAT (SLC6A3) and in vivo striatal DAT availability in the human brain [58], the findings from SPECT studies concerning its association with striatal DAT availability appear not to be consistent [59]. 

## 4. DAT Imaging and Its Relationship to the Pathological Degeneration of the Nigrostriatal Pathway

In line with the characteristic motor asymmetry of PD, it is usual to see asymmetric DAT loss on functional imaging being more marked in the hemisphere contralateral to the most affected side of the body [60]. The asymmetry of dopaminergic depletion, detected by DAT imaging, has been also directly related to the magnitude of dopaminergic response [61]. Some authors suggested increased “left hemisphere susceptibility” in PD, in that the left nigrostriatal pathway is more affected than the right [62], which has been interpreted as an effect of handedness. This is consistent with several studies showing greater proportions of right-handed PD patients presenting with greater motor impairment of their right compared with their left-sided limbs [63]. However, a proportion of PD patients present their primary symptoms on the side of the body ipsilateral to the predominant dopamine deficit, and this appears to be common even in patients with clear motor and DAT asymmetries, arguing against hemispheric dominance as the only factor determining asymmetric nigrostriatal dysfunction in PD [64]. As clinical asymmetry becomes less prominent over the course of the illness, as there is a progressive loss of side-to-side DAT asymmetry with advancing disease [65]. In fact the decline in DAT binding seems to occur at a similar rate across striatal subregions, but maintains the antero-posterior gradient of dopamine dysfunction which is highly preserved during disease course [66]. This may be the result of the different vulnerability of nigrostriatal connections in PD, with an early and prevalent involvement of dopaminergic neurons of the ventrolateral tier of the substantia nigra pars compacta (SNpc) projecting to the dorsal putamen, as suggested by pathological studies [67]. Conversely, a different striatal gradient, with relatively greater caudate involvement, has been observed in patients both with dementia with Lewy bodies (DLB) and Parkinson dementia (PDD) vs. PD, in keeping with the hypothesis that reduced striatal DAT availability in the caudate nucleus is associated with a worse cognitive performance [68,69]. Interestingly, over the course of PD the change appears to be greater in the putamen ipsilateral to the predominant dopaminergic deficit, when compared with the contralateral putamen, suggesting a potential “floor effect” in the latter [70]. However, the putamen remains more affected than the caudate and, according to a PET study with [^18^F]FP-CIT, the posterior putamen is the subregion exhibiting the fastest annual rate of decline with disease progression [71].

It is worth mentioning that differences in the type of radiotracers, sample sizes, study design, participant characteristics and other methodological differences may be reason for discrepancies in imaging studies. Nevertheless, DAT-SPECT can be an excellent tool to map the spatial and temporal patterns of dopaminergic dysfunction in PD, providing evidence of a negative exponential loss of dopaminergic cells in early disease, which slows down with increasing symptom duration [72]. Clinical features seem to follow a similar exponential model with a faster progression early in the disease than in later years [73]. Accordingly, pathologic data have suggested an early and substantial reduction in dopamine function soon after diagnosis, but with a virtual absence of fibers in the dorsal striatum at 4 years [74]. Yet, imaging studies indicate that approximately 50% of putaminal presynaptic dopamine function is still preserved in PD patients with a mean motor disease duration of 4 to 7 years [75]. Moreover, residual functional dopaminergic nerve terminals have been demonstrated even in the striatum of PD patients with disease durations of more than 15 years [76]. Compensatory mechanisms in the terminal area of the nigrostriatal tract in PD could explain this striking discordance between neuropathology and neuroimaging data. It is acknowledged that motor PD symptoms appear when there is about a 30% loss of total dopaminergic neurons in the SNpc and striatal dopamine content is approximately reduced of 80% [77]. The magnitude of dopaminergic cell loss and striatal dopamine depletion before PD motor symptoms supports the key role of adaptive mechanisms which may effectively counteract nigrostriatal dysfunction at the onset of cellular dysfunction. Among the striatal regulatory changes occurring to partially compensate for the loss of dopaminergic terminals, DAT function has demonstrated to be downregulated to maintain the basal ganglia output within normal limits in response to the reduced levels of synaptic dopamine [78]. Interestingly, extra-striatal processes of neuroplasticity and the increased serotonergic fiber density with its capacity for dopamine release within the denervated striatum may constitute additional adaptive mechanisms of compensation [79]. Additionally, recent evidence on mice models suggests that striatal dopamine is synthesized not only in dopaminergic nigrostriatal axons, but also in the so-called monoenzymatic neurons containing complementary enzymes of DA synthesis, contributing to compensation of the dopamine deficit in the degraded parkinsonian striatum [80]. It is conceivable that compensatory molecular strategies are more pronounced in the pre-symptomatic phase of the disease, when most of the damage to the nigrostriatal dopamine pathway seems to occur [81]. In keeping with this, in asymptomatic mutation LRRK2 carriers, DAT reduction was found to be more pronounced than changes in VMAT2 and L-AADC indicating in vivo a strong involvement of compensatory DAT down-regulation in the pre-symptomatic stage of PD aiming to delay onset of clinical symptoms [82]. A significant reduction in striatal DAT binding but a preserved nigral signal measured with 7T Magnetic Resonance Imaging (MRI) has been observed in a still asymptomatic member of a family carrying the same G2019S LRRK2 gene mutation, supporting possibly compensatory down-regulation of DAT in the premotor disease stage [83]. Still, there are indications that early compensatory mechanisms are more efficient in younger brains [84]. This could argue in favor of a relatively long pre-clinical period in early onset PD patients, but also their greater propensity towards motor response complications, in line with a different time course of regulatory changes as a function of disease progression [85]. To put it another way, compensatory early events could have long-term, deleterious effects on basal ganglia function, ultimately contributing to the development of motor fluctuations and dyskinesias in PD [86]. This could be the effect of an increased dopamine turnover in the presence of reduced DAT levels because of advanced PD, with greater oscillations in L-dopa derived synaptic DA which have been linked to the occurrence of motor response complications [87]. In this regard, we recently found lower putaminal DAT binding and a higher rates of motor complications in early onset de novo PD patients despite a less severe motor phenotype at baseline than those with late onset PD, supporting an age-dependent different functional significance of DAT in early disease [88]. Adaptive striatal changes are greater in the initial stage of disease, especially in the putamen, suggesting that the eventual onset of motor symptoms in PD may reflect a global failure of compensatory mechanisms in nigroputaminal dopaminergic neurons [89]. As a consequence, it is not surprising that motor signs appear between a loss of 56 and 71% of DAT ligand, due to striatal compensatory adjustments as well as high nigrostriatal reserve [90]. In addition, although the severity of cardinal motor symptoms, assessed by the motor section of the unified Parkinson’s disease rating scale (UPDRS), is considered a good predictor of the neuronal loss observed in the substantia nigra (SN) [77], several studies attempting to correlate DAT in vivo imaging outcomes and neuropathological findings in humans have provided conflicting results [91]. Nigral neuronal density was found to be a significant predictor of DAT uptake in the striatum, as measured with [^123^I]FP-CIT SPECT, in a group of patients comprising of autopsy-confirmed cases with Alzheimer’s disease (AD), DLB and PDD [92]. Again, a high correlation between in vivo striatal DAT binding and dopaminergic cell counts in the SN at autopsy was detected in a heterogeneous cohort of patients with clinically suspected parkinsonism, even in those with moderate to severe dopaminergic degeneration [93].

Conversely, there is evidence in animal models of PD that striatal DAT uptake strongly correlates with striatal dopamine levels but does not faithfully reflect loss of nigral neurons throughout the full range of neuronal loss [94]. Notably, in a multitracer PET study in 1-methyl-4-phenyl-1,2,3,6-tetrahydropyridine (MPTP)-treated monkeys, striatal uptake correlated with stereological nigral cell counts only with cell loss <50%, with a flooring effect in moderate to severe parkinsonism [95]. In keeping with this, recent functional imaging studies raised important questions about the interpretation of DAT-SPECT in PD demonstrating a lack of correlation between striatal dopaminergic innervation and antemortem DAT imaging [96,97]. If the striatum is the initial site of nigrostriatal damage in PD, we could argue that there could be a positive relationship between striatal DAT binding and the number of axons of the nigrostriatal pathway, considering even the possibility that reduced DAT expression may reflect dysfunctional but viable nigral neurons in PD. However, Honkanen and colleagues failed to show a correlation between putaminal DAT binding and the number of dopaminergic axon counts in 14 patients with neuropathologically confirmed PD or atypical parkinsonism [97]. Taken together, these results tentatively suggest that DAT imaging is not a surrogate marker of PD pathology but rather a functional measurement of nigrostriatal dopaminergic pathway, reflecting axonal activity or DAT expression [98]. However, caution must be applied to such findings, performed in small number of patients and complicated by the time interval between scanning and cell counting which is an intrinsic critical issue of any clinicopathological post-mortem study [99]. Indeed, it is important to note that results might be different in early PD patients in which striatal DAT seems to correlate with the severity of parkinsonism. A potential approach to overcome the limitations associated with the time-interval between the in vivo scan and the post-mortem examination might be to correlate in vivo dopaminergic neuromelanin-rich neurons in SN measured by MRI with striatal DAT levels [100]. Indeed neuromelanin-sensitive MRI (NM-MRI) sequences have demonstrated the ability to be a potential in vivo index of nigral cell death [101]. First multimodal in vivo imaging studies showed a good correlation between NM-MRI SN measures and striatal DAT-SPECT values both in cohorts of patients with PD and mixed parkinsonism [102,103]. However, Martìn-Bastida and colleagues detected a moderate to strong linear association between the two measures across SN and striatum only in the clinically-defined most affected side of moderate stage PD patients, supporting an early asynchrony in the loss of neuromelanin-containing versus tyrosine hydroxylase positive nigral cells [104]. In addition, no correlation was found between NM-MRI in the SN and striatal binding ratio of DAT in another sample of PD patients, supporting the role of DAT-SPECT for early PD diagnosis whereas NM-MRI appears to be useful for determining dopaminergic neuronal degeneration in advanced PD [105]. Hence, the linear correlation between the severity of the parkinsonism and the loss of dopaminergic neurons indicates that molecular imaging markers that reflect residual nigral neuronal counts might provide a more suitable biomarker of clinical progression in advanced parkinsonism [106]. This has been demonstrated in MPTP-treated primates, by comparison of measured binding potential for DAT ligand in the SN with quantitative cell counts in the nigra, confirming that nigral specific binding of a DAT marker clearly reflects the number of dopaminergic neurons [95]. Accordingly, a recent PET study examining in vivo DAT availability along the entire dopaminergic nigrostriatal system using [^18^F]FE-PE2I, found a 30% of DAT loss at the level of the nigral cell bodies at disease onset, in line with pathologic data about the degree of dopaminergic neuron loss in the SN [107]. A substantial reduction of presynaptic DAT availability in the dorsal putamen and caudate, as opposed to a limited reduction in the SN, was found in another imaging study by Caminiti and colleagues, on patients with early-stage idiopathic PD [108]. This supports the notion that loss of striatal axonal markers of dopamine neurons exceeds the loss of their nigral cell bodies consistent with the apparent retrograde degeneration affecting the nigrostriatal pathway in PD [109,110]. This is not surprising, because axonal loss is now recognized as an early and predominant feature of PD, further corroborated by the evidence that α-synuclein pathology is more abundant in axons and presynaptic terminals [111,112].

## 5. DAT Imaging as Diagnostic Biomarker

Although several ligands have been produced for DAT visualization through SPECT and PET imaging, Ioflupane or [^123^I]-FP-CIT is the only approved in vivo diagnostic imaging agent for clinical practice [113]. Notably, DAT-SPECT is indicated in patients with clinically uncertain parkinsonian syndromes, with high sensitivity concerning the diagnosis of PD and a significant effect in influencing both further investigations and therapeutic intervention [114]. The clinical utility of DaTSCAN was investigated in a recent systematic review and meta-analysis demonstrating that the results of DaT imaging are associated both with a change in diagnosis and management of patients with suspected parkinsonian syndromes, even in individuals with long symptom duration [115]. However, despite a rigorous clinical assessment, diagnosing de novo untreated PD can be challenging, even for movement disorders specialist [116]. For example, the clinical presentation of isolated or atypical tremors can be insufficient to allow a precise early-stage diagnosis whereas the detection of abnormal striatal DAT binding in such patients predicts the future development of additional signs and symptoms consistent with clinical criteria for PD [117]. On the other side, patients with essential tremor (ET) were found to have mild striatal DAT abnormalities than healthy controls, but not as low as those with PD and with a pattern of DAT loss different from PD involving both caudate nucleus and putamen [118]. In the three-year follow-up study of the same cohort, no loss of DAT uptake was reported but ET patients continued to exhibit a predominant impairment of DAT in the caudate, suggesting the selective dopaminergic loss in the caudate nucleus as a possible pathophysiological link with PD. However, DAT-SPECT imaging allows differential diagnosis between ET and PD with a specificity comprised between 97 and 100% [119]. Importantly, an Italian study reported an overall decrease of costs for the health care system and an increase of patients’ time on potentially beneficial therapy by implementing the use of [^123^I]-FP-CIT SPECT in the early differential diagnosis of ET and PD [120].

Additionally, in several studies DAT-SPECT demonstrated a high accuracy in differentiation between PD from non-degenerative parkinsonisms such as those secondary to vascular, toxic, and inflammatory processes, or that are drug induced [121]. However, if DAT imaging in pure vascular parkinsonism is typically normal, other reports have described an involvement of the nigrostriatal system in about two-thirds of patients with parkinsonism and cerebrovascular disease [122]. Striatal asymmetric index is usually significantly higher in PD compared with VP, although occasionally a pattern similar to that described in PD can be found [123]. Vascular lesions that acutely or subacutely affect the basal ganglia may produce a significant pre-synaptic dopaminergic deficit congruent with the focal stroke location [124]. A DAT deficit has been also observed in cases with multiple and diffuse subcortical and periventricular white matter lesions indicating that symptoms of parkinsonism in some patients with diffuse leukoaraiosis can be related to a detectable nigrostriatal degeneration [125]. Thus, striatal DAT assessment may help identifying a comorbid nigrostriatal dopaminergic denervation in doubtful cases with vascular parkinsonism, and this can have therapeutic implications selecting patients who are more likely to response to levodopa [126].

Similarly, DAT imaging is expected to be normal in drug-induced parkinsonism (DIP), a relatively common postsynaptic parkinsonism in people chronically exposed to D2-receptor blocking agents and which can be clinically indistinguishable from PD [127]. Nevertheless, a subtle to severe involvement of the nigrostriatal system is found in up to 50% of patients with DIP, consistent with a progression of motor disability reported in a subset of DIP cases despite withdrawal of the offending agent [128,129]. Accordingly, imaging studies have confirmed DAT abnormalities in a number of patients clinically diagnosed with DIP, in keeping with a concomitant degenerative process, deemed to reflect PD or a subclinical form of the disease unmasked by the anti-dopaminergic drugs [130]. In this regard, a “double hit” hypothesis has been proposed, invoking a primary neurotoxic effect of dopamine blocking agents [131] and autopsy studies have reported changes compatible with an underlying idiopathic PD [132]. Moreover, there are patients with persistent DIP but visually normal DAT imaging in which mechanisms through which the offending drugs lead to parkinsonian symptoms are yet unclear, but a relatively decreased DAT levels within normal values could be indicative of a subtle nigrostriatal dopaminergic dysfunction predicting an early development of DIP [133,134]. This supports the evidence that DIP diagnosis represents a risk factor for idiopathic PD, implying the existence a latent parkinsonism in several patients triggered by the causative drug [135].

It is worth mentioning that DAT imaging has also a recognized role in distinguishing DLB from other forms of dementia, mainly AD, in uncertain cases [136,137], with a sensitivity up to 100% by using the semi-quantitative approach [138]. However, dopaminergic imaging cannot distinguish DLB from PDD, as both are associated with nigrostriatal dopaminergic neurodegeneration [139] or from patients with other dementia types (e.g., frontotemporal dementia), but having variable degrees of parkinsonism [140,141]. Nonetheless, not all patients with DLB have a positive scan, potentially becoming abnormal later in the disease process in a model of rostral-to-caudal progression pattern of Lewy body pathology instead of the reverse, as is usual [142]. Similarly, DAT-SPECT imaging was found to be normal in about 10% of cases of a large cohort of patients with clinical diagnosis of corticobasal syndrome (CBS), despite prominent bilateral extrapyramidal signs [143]. However, if the presynaptic nigrostriatal function may be preserved in the early stages, a progressive decline of striatal binding was demonstrated on follow up imaging [144] as well as confirmed pathologically [145].

Moreover, there are several other clinical scenarios where DAT imaging may be useful, such as in patients with mild or inconsistent parkinsonism despite detailed examination, or patients with parkinsonism but suboptimal response to levodopa [146,147].

Nevertheless, although DAT imaging offers an extraordinarily valuable tool in detecting the presence of a dopaminergic degenerative process, it has to be interpreted just as a useful adjunct to clinical decision making and cannot replace the benefits of thoughtful clinical evaluation [148] Indeed, abnormal DAT scan increases with increasing clinical probability of PD and number of cardinal motor signs present [149]. Older age, motor symptom asymmetry, and shorter disease duration have been identified as possible factors associated with abnormal scans in a large and heterogeneous sample of patients with clinically uncertain parkinsonism or suspected PD [150]

Moreover, it is important to note that an abnormal DAT scan does not necessarily indicate a diagnosis of PD. Indeed, specificity of DAT-SPECT is rather low, and it cannot reliably distinguish PD from atypical parkinsonisms (progressive supranuclear palsy-PSP, multiple system atrophy-MSA and CBS) in which there is a loss of striatonigral dopamine neurons as in PD. Some attempts have been performed to use quantitative DAT-SPECT measures, such as the asymmetric index and the caudate/putamen ratio, for a differential diagnosis between PD and atypical parkinsonisms, but without consistent results for their application in clinical practice [151]. In early PD the DAT signal appears typically most depressed in the posterior putamen contralateral to clinically affected limbs whereas the head of caudate and ventral striatum are relatively preserved [152]. Hence, in visual assessments, egg-shaped pattern has been reportedly more indicative for PD whereas a burst striatum pattern is more common in patients with a clinical diagnosis of atypical parkinsonism. 

To further complicate matters, a normal scan could not necessarily exclude a diagnosis of PD. In drug trials of early PD using DAT imaging to enroll patients, 5.7–14.7% of the cases clinically diagnosed as PD had “scans without evidence of dopaminergic deficit” (SWEDD) [41,42,153]. In the majority of these subjects, follow-up scan remained normal in the context of minimal evidence of clinical progression. Thus, it is very likely that most patients with SWEDD, presenting with subtle atypical features, do not have PD but lay mainly between dystonic tremor, ET and other conditions mimicking features of parkinsonism [154,155]. Accordingly, longitudinal observational studies seem to support the notion that SWEDD may simply be a misdiagnosis of other different conditions [156]. It should be also noted that patients could be initially misclassified as SWEDD for an incorrect visual interpretation of DAT imaging [19,157], even though visual assessment of DAT-SPECT images has been established as being highly reliable in the identification of degenerative parkinsonisms [158].

However, some patients with parkinsonism and normal DAT imaging have been reported to both have abnormal scans on follow-up and respond well to dopaminergic therapy [159,160], raising the possibility that DAT-SPECT can be normal in the very early phase of PD [161,162] and challenging the concept of normal DAT-SPECT as an absolute exclusion criterion [6]. Besides, some SWEDD patients may have genetic forms of PD [163], further indicating that a negative DAT-SPECT should not definitively rule out the diagnosis of PD in an individual case [164]. In addition, other data (mainly derived from patients included in the Parkinson’s Progression Marker Initiative study-PPMI) suggest that subjects with SWEDD have an increased burden of non-motor symptoms, both in relation to frequency and severity, compared to PD subjects [165,166,167,168,169]. Conversely, hyposmia is more common in PD but not all patients with SWEDD have normal range in olfactory function [170]. As a consequence, some authors support the notion that patients with SWEDD represent a group distinct from PD but not free from pathology [154,169,171]. Thus, SWEDD remains a heterogeneous and highly debated phenomenon [162].

Use of DAT imaging has been suggested as enrichment biomarker for patient selection in early PD clinical trials for eliminating subjects without apparent striatal dopamine nerve terminal loss and thus allowing reduction of trial size [172]. DAT imaging has confirmed its diagnostic accuracy in a recent paper on PPMI patients, in which the authors found a low rate of diagnostic revision (2%) in early parkinsonism when DAT imaging was utilized, as opposed to a change of 6–13% in diagnosis reported in several PD clinical trials which did not consider DAT binding deficit as an inclusionary criterion [173]. However, while exclusion of SWEDD subjects from trials will improve the chance of determining clinical benefit of disease modifying treatment, some SWEDD subjects who demonstrated to experience a clinically important worsening of the motor scores would be excluded in a DAT-based enriched trial [174].

## 6. DAT Imaging as Preclinical Biomarker

In PD pathological processes insidiously progress and motor deficits generally appear when 50–60% of dopaminergic neurons in the SN are already lost and striatal dopamine is depleted by 80% [175]. This suggests a potential window for detecting loss of DAT binding in subjects who have subclinical dysfunction, identifying pre-symptomatic nigrostriatal dysfunction in at-risk subjects. Hence, dopamine deficiency has been shown by DAT imaging in individuals with conditions associated with increased risk of future PD such as hyposmia, rapid-eye-movement (REM) sleep behavior disorder (RBD) [176] or particular genetic conditions [177]. Although DTBZ binding provides the earlier and most reliable estimate of dopamine terminal density, actually DAT binding appears to be another sensitive marker of early disease, presumably attributable to a combination of reduced DAT binding sites with loss of dopamine nerve terminals as well as down-regulation of DAT in surviving terminals [178]. Several studies indicate that idiopathic RBD (iRBD) is an early feature of α-synucleinopathy, with the majority of patients who will eventually develop PD and DLB if they live long enough [179]. 

Reduced striatal DAT signaling has been reported in about 50% of subjects with isolated RBD [180]. Interestingly, in a prospective study on a large iRBD cohort, the authors showed dopaminergic imaging abnormalities in subjects at increased short-term risk for development of PD, similar to those typically seen in PD, with a tracer uptake reduction more pronounced in the putamen than in the caudate nucleus [181]. Additionally, a progressive decline in striatal binding in patients with iRBD further support the notion that RBD is actually an integral prodromal period of a defined neurodegenerative syndrome [182]. Several recent studies confirmed the high sensitivity of abnormal DAT-SPECT to accurately predict the short-term conversion to a clinically-defined α-synucleinopathy [183,184,185,186,187]. 

In the same vein, dopaminergic imaging could be also used to identify a high-risk group in subjects with hyposmia, which is another common prodromal feature of PD [188,189]. Abnormal DAT findings were found in 11% of hyposmic subjects only [190], but longitudinal studies of striatal DAT imaging in patients with hyposmia have shown a high risk of PD phenoconversion within 4 years [191]. Hyposmic individuals with DAT deficits also converted to PD at a higher rate that hyposmic individuals without a DAT deficit [191]. Recently, long-term follow-up of the PARS (Parkinson Associated Risk Syndrome) cohort confirmed baseline DAT as a strong predictor of conversion to clinical PD for persons with hyposmia and a DAT deficit [192]. Moreover, a progression of DAT deficit was also observed, with an incident DAT deficit in subjects who had either indeterminate or normal DAT status at baseline. Image conversion was also predictive of conversion to motor PD [192].

In support of the role of DAT imaging as marker of nigrostriatal dopamine denervation in the prodromal phase of the disease, Noyce and others investigated whether DAT abnormalities were also present in individuals with other prodromal features of PD [193]. In addition to hyposmia and RBD, they reported an association of striatal binding ratio with subtle motor dysfunction and PREDICT-PD risk estimates, which combine a number of risk and pre-diagnostic features of PD [193] Consistent with this report, elderly individuals with minimal parkinsonism without PD exhibited mild decrease in DAT availability in the striatum, which may be related to the disease process of prodromal PD [194].

Subclinical nigrostriatal DA dysfunction has been previously demonstrated in subjects with a high genetic risk of PD [195,196]. Decreased putaminal DAT binding has been reported in one unaffected individual heterozygous for the α-synuclein mutation [197]. As mentioned before, clinically unaffected mutation LRRK2 carriers can have reduced DAT binding, in keeping with active compensatory mechanisms at an early preclinical stage [82]. Similarly, in a multitracer PET study undertaken by Wyle and colleagues, DAT binding in LRRK2 mutation carriers without PD was significantly lower than in healthy controls even in younger age groups, by contrast with findings of ^18^F-FDOPA uptake which was preserved until age 70 years [163]. Converging results were obtained in other studies, with lower DAT levels in asymptomatic carriers of LLRK2 mutation than in noncarriers [83,198,199,200]. The presence of nigrostriatal dopaminergic denervation was found to be larger in the putamen than in the caudate in a cohort of asymptomatic relatives of patients carrying the LRRK2 R1441G mutation, reflecting the pattern of dopaminergic denervation observed in patients with PD. In such subjects reduced putaminal DAT density was even associated with a poorer execution of demanding timed motor tests [201]. It is important to note that penetrance estimations of LRRK2 mutations vary widely and only a proportion of mutation carriers will develop motor symptoms of PD [202]. The baseline DAT binding showed a high sensitivity and specificity in predicting conversion to motor PD within the 4-year period in a recent longitudinal study on 29 carriers of the G2019S LRRK2 mutation [203]. Although few reports are available for non-manifesting carriers of glucocerebrosidase (GBA) mutations, 3% of GBA non-manifesting carriers enrolled in the PPMI cohort showed a DAT deficit at baseline DAT-SPECT [204]. Interestingly, non-manifesting carriers of GBA mutation, but not LRRK2 non-manifest mutation carriers, had increased striatal binding values in all striatal regions when compared with LRRK2 mutant carriers and healthy controls, leading the authors to hypothesize a role of compensatory mechanisms [205]. However, a downregulation of DAT would be the expected response that serve to maintain extracellular levels of dopamine, calling also on alternative explanations [206].

## 7. DAT Imaging as Predictive Biomarker: Motor Measures

Discovering meaningful endpoints that measure progression is one of the greatest challenges in PD therapeutics. With continuing efforts and advances in the field, imaging strategies hold promise in identifying potential molecular targets of relevance to disease-modifying agents for PD. However, the prognostic validity of DAT-SPECT concerning the clinical progression of PD is unclear and potential confounding factors include a slow disease progression, antiparkinsonian medications, compensatory changes and aging. It should also be taken into account that several clinical patterns of decline exist for different subtypes of PD and the rate of decline is probably slower in young PD patients, as confirmed by longitudinal dopaminergic imaging studies [84]. Furthermore, we need to consider that commonly used radioligands for PET and SPECT also have an affinity for the serotonin (SERT) and noradrenaline (NET) transporters [207]. Nevertheless, in early cross-sectional studies of PD cohorts, a lower baseline DAT binding correlated with the increasing disease duration and motor severity measured by the UPDRS and the Hoehn and Yahr stadium [11,208,209,210] (Table 1). However, a better correlation of DAT binding with bradykinesia and rigidity than with tremor is extensively reported, supporting an independent pathophysiology for tremor which is not assessed by DAT-SPECT techniques [211]. Indeed, PD patients with a tremor-dominant type tend to show a significantly higher putaminal DAT uptake than akinetic-rigid and postural instability-gait disorders phenotypes at the same stage of disease [211,212,213], suggesting different patterns of dopaminergic degeneration in different subtypes of PD [214]. In keeping with this, the visual inspection of DAT-SPECT images can reveal significantly different morphological patterns for tremor-dominant (“eagle-wing shape”) versus akinetic-rigid (“egg-shaped”) PD patients, allowing a reliable differentiation of clinical PD subtypes [215]. Nonetheless, semiquantitative analysis of FP-CIT scan found no differences between the two groups [215]. There is some evidence that dopamine depletion in nuclei other than the striatum (i.e., the retrorubral area) may play a role in parkinsonian tremor [216]. In addition, PD tremor could be related to dysfunction of the serotonergic system in areas related to motor function. Accordingly, reduced [^123^I]-FP-CIT binding in the brainstem raphe nuclei, reflecting serotonin transporter availability, correlated with tremor scores in a subgroup of early PD patients with a tremulous motor phenotype [217]. Similarly to tremor, postural and gait disturbances were strongly correlated with cholinergic rather than with nigrostriatal dopaminergic activity [218]. Although the mechanisms underlying freezing of gat (FOG) have not yet been identified, recent retrospective studies conducted in early [76] or de novo PD patients [219] have found that the degree of presynaptic dopamine depletion, examined by DAT uptake, predicted the subsequent development of this phenomenon. There are conflicting results about which striatal subregion is more closely linked with FOG, but reduced DAT uptake in the caudate nucleus correlated with the development of FOG in early PD patients [220].

Recently, Mäkinen and colleagues investigated the associations of individual parkinsonian motor signs with striatal dopamine deficiency in patients with clinical parkinsonism or tremor of an uncertain origin, finding the highest likelihood of DAT deficiency in upper extremity rigidity and hypomimia [221].

Ravina and colleagues investigated whether baseline DAT levels could predict the development of clinically important long-term motor and nonmotor outcomes. Subjects with lower striatal DAT binding within 2 years of the clinical diagnosis of PD were more likely to develop motor-related disability, as well as cognitive impairment, psychosis, and depression after more than 5 years of follow-up [43]. In this retrospective study, the potential prognostic value of DAT imaging seems to be limited to the baseline scans because only a small association with long-term clinical outcomes was found for the DAT rate changes based on the follow-up scans, in line with a slower rate of nigral degeneration observed over the course of the disease [74].

In contrast, more recent longitudinal studies have failed to demonstrate a correlation between change in the mean striatum uptake and the change in UPDRS motor score [222]. Latourelle and colleagues did not find a predictive effect of DAT-SCAN imaging data on motor progression in their study based on previously established and potential novel markers by using an unbiased machine-learning approach [223]. In a longitudinal study of 44 PD followed-up for a mean of 44 months, baseline ß-CIT SPECT data were not in accordance with the decline of clinical symptoms over the observation period, even though inversely correlated with the UPDRS motor score at baseline [224]. Still, the observed outcome discrepancies between clinical and imaging changes in comparative drug trials of levodopa vs. dopamine agonists using β-CIT SPECT and ^18^F-dopa PET, could be the effect of a poor significant predictive value of baseline dopaminergic imaging, even though we have to consider potential differential influence of antiparkinsonian drugs on radiotracer uptake [41,42]. Similarly, there was a discordance between dopaminergic PET measures, namely ^18^F-DOPA, and clinical outcomes in trials of fetal nigral grafting and intraputaminal glial cell line-derived neurotrophic factor (GDNF) in PD patients [225,226]. In a cross-sectional study conducted in a population extrapolated from the PPMI dataset, significant correlation between DAT values and clinical measures, including the UPDRS-III and disease duration, was found only when both healthy controls and PD patients were included in the analysis whereas this relationship failed to remain significant after taking out healthy controls [227]. Similarly, 5-year longitudinal data on the change of clinical and DAT imaging outcomes measures in early, untreated PD patients from the PPMI cohort showed only a weak correlation between MDS-UPDRS-III and DAT binding, most marked at baseline, but no correlation between the rate of change of the 2 variables [70]. In keeping with this, recently no relationship was found between striatal or extrastriatal DAT binding and survival in PD patients [228], but other important neuropathological events and non-dopaminergic mechanisms could be more relevant in predicting also mortality [229]. A recent meta-regression analysis uncovered linear correlation between caudate DAT binding and disease severity in PD as studied cross-sectionally, likely due to earlier and more severe putaminal dopaminergic defect [75]. As described above, the important flooring effect found in the decline pattern of putaminal DAT can explain the lack of correlation with clinical scores, indicating the suboptimal value of striatal presynaptic dopaminergic functional imaging as disease progression marker [94].

Conversely, in a recent PET study using ^11^C-PE2I (a radioligand with high striatal binding and greater DAT specificity than the others), the authors found not only a high correlation between the reduction in striatal DAT density and PD symptom severity at baseline, but even a significant negative correlation between change in motor severity (both UPDRS-III and bradykinesia/rigidity subscores) and DAT binding in the caudate and posterior putamen [184,230]. Of particular interest is that even the most affected putamen confirmed a relationship at follow-up between changes in ^11^C-PE2I and in bradykinesia-rigidity scores. This study suggests that DAT quantification using DAT ligands with relatively high DAT-to-SERT selectivity could be more sensitive for investigating disease progression [184]. Furthermore, use of radiomic features extracted from DAT-SPECT images or the combination of multi-parametric and multi-modality solutions, involving different MRI sequences and/or SPECT images, along with non-imaging features, may significantly improve prediction of motor outcomes [231,232].

## 8. DAT Imaging as Predictive Biomarker: Motor Complications

There is ample evidence to point that striatal dopamine depletion, particularly in the putamen, whether due to neuronal degeneration or functional impairment, predicts the development of motor complications in patients with PD [243,244,245] (Table 1). Specifically, lower levels of baseline DAT activity in the posterior putamen were found to be a strong and independent predictor of the development of levodopa-induced dyskinesia (LID) at the 3-year follow-up in 127 drug-naive de novo patients [87]. Initial DAT activity in the posterior putamen was also significantly associated with the early appearance of LID in another retrospective cohort study by the same authors including a total of 412 consecutive drug-naive patients with PD [233]. Beyond the initial absolute DAT values, baseline and follow-up [I^123^]-FP-CIT SPECT images from the PPMI population demonstrated that even the deterioration rate of putaminal innervation was significantly higher in PD patients who developed LID during 48 months of follow-up [234]. Similarly, time-related changes in striatal DAT availability have been related to the appearance of future LID in a small longitudinal SPECT study of Piccini’s group [246]. In another large number of patients with de novo PD, subjects with wearing-off (W-O) exhibited lower level of DAT activity in the anterior putamen than did those without W-O [235]. As additional evidence of the regional difference between W-O and LID, a recent study by using a factor analysis based on the DAT availability in striatum, found a relationship between the posterior putamen, which belongs to the sensorimotor striatum, and a higher risk for LID, as well as an association between dopamine depletion in the anterior putamen and both early development of W-O and conversion to dementia [237]. Therefore, patterns of striatal dopaminergic denervation seem to have prognostic implications in patients with early-stage PD [237]. 

The exact mechanisms of motor response complications are unclear, but DAT reduction would converge to disrupt presynaptic dopamine homeostasis and increase oscillating levels of synaptic dopamine in the parkinsonian striatum, ultimately facilitating the appearance of LID and motor fluctuations [247]. Additionally, repeated administrations of levodopa with chronic pulsatile stimulation of dopamine receptors, and postsynaptic changes including dopamine receptor supersensitivity, may play together an important role in their pathogenesis. While young age at onset is a well-known risk factor for developing LID, the reasons underlying such high risk have not been resolved. In this regard, we recently proposed that a different availability of DAT between PD patients with young and old onset, as the result of age-related differences in early striatal compensatory mechanisms, could account, at least in part, for age-at-onset–dependent risk of motor complications in PD [88]. Therefore, early compensatory DAT downregulation in younger PD patients could be a relevant susceptibility factor to motor complications throughout the course of disease. 

Notwithstanding, although there is strong support to the presynaptic hypothesis of motor complications, other studies failed to demonstrate a relationship between DAT binding on SPECT in early-stage PD and their late emergence [76,236]. It is important to note that, alongside these dopaminergic mechanisms, increasing evidence suggests a direct involvement of glutamatergic, serotonergic and cholinergic system in the expression of dyskinesias [248,249]. Notably, striatal serotonin hyperinnervation appears to be a key determinant in the appearance of LID, as revealed by imaging studies demonstrating a strong correlation between the SERT to DAT ratio, as reflected by the ^11^C-DASB BP to ^123^I-ioflupane uptake ratio, and LID [45,250]. 

## 9. DAT Imaging as Predictive Biomarker: Non-Motor Measures

Several DAT imaging studies revealed an association between decreased striatal DAT availability and non-motor symptoms in PD including anxiety [251], depression [252,253,254], apathy [255], impulsivity [239,256], fatigue, constipation [240], hyposmia [257,258,259,260], autonomic symptoms [261,262,263,264,265,266], sleep disturbances, daytime sleepiness [267], body weight loss [268] and visual hallucinations [241], in support of a partial dopaminergic pathogenesis [269] (Table 1). In a recent study using data from sequential DAT-SPECT imaging in 344 PD patients from the PPMI dataset, Liu and colleagues demonstrated a significant association between DAT binding both at baseline and at follow-up scans and several baseline non-motor symptoms, with RBD showing the strongest association with concurrent and future DAT binding [270]. Moreover, the burden of non-motor symptoms has been associated with distinct patterns of striatal dopamine depletion in a large cohort of patients with de novo PD [271].

However, other findings are dissimilar and there are only few longitudinal data addressing the prognostic impact of striatal DAT on non-motor outcomes, suggesting that dysregulation in other neurotransmitter systems is an important contributor to their pathophysiology [272,273,274,275,276,277,278,279]. 

Conversely, it is well known the dopaminergic contribution to cognition, mainly in frontal/executive performance, and empirical evidence for this hypothesis comes from several DAT imaging studies reporting a significant relationship between striatal dopaminergic denervation, especially in the caudate, and cognitive functioning [280,281,282,283], both in de novo [284] and advanced PD patients [285]. The occurrence of early caudate dysfunction has been related to later development of non-motor symptoms, such as RBD, depression and cognitive impairment, with overall worse prognosis [286,287]. Furthermore, recent studies targeted the clinical relevance of extrastriatal DAT uptake in early PD, identifying a significant contribution of the DAT uptake within posterior cortical areas to global cognitive status [288]. A recent study from the large PPMI dataset has investigated the role of asymmetric DAT loss as a potential biomarker in drug-naive PD patients followed longitudinally up 4 years revealing a specific influence of hemispheric asymmetry in dopaminergic cell loss on both motor and cognitive outcomes in PD [289].

## 10. Beyond DAT: Correlation with Other Potential Nigrostriatal Biomarkers in PD

It should be mentioned that other tools and markers have been recognized as useful early diagnostic biomarkers in PD. Novel MRI techniques, able to assess nigral pathology, hold the potential for the differentiation of neurodegenerative from non-neurodegenerative parkinsonian disorders, but may also enhance subtyping and disease severity monitoring in PD [290].

Visual assessment of dorsolateral nigral hyperintensity (DNH) on iron-sensitive MRI sequences yields excellent diagnostic accuracy (pooled sensitivity of 98% and a pooled specificity of 95%) for PD versus healthy controls [291]. Furthermore, absence of DNH on high-field susceptibility weighted imaging (SWI) has also been suggested as a potential MRI biomarker in prodromal degenerative parkinsonism since it has been found in clinically asymptomatic LRKK2 carriers [83] and in at least two-thirds of subjects with iRBD [292]. Accordingly, a good positive correlation between nigrosome imaging and DAT SPECT imaging on both 3- and 7-T MRI has been reported [293]. However, there is some degree of discordance between findings at MRI imaging and ^[123]^I-FP-CIT SPECT, reinforcing the notion that nigrostriatal degeneration occurs before nigral cell loss [294].

Early PD diagnosis (differential diagnosis from healthy controls) using NM-sensitive MRI has a sensitivity of 89% and a specificity of 85% [295]. Again, a significant positive correlation between the NM-positive SN volume on NM-sensitive MRI scans and ^[123]^I-FP-CIT uptake ratios on SPECT scans has been demonstrated [296]. Importantly, multimodal studies employing structural MRI and SPECT imaging showed comparable or better diagnostic performance compared to each parameter of DAT-SPECT and NM-MRI in distinguishing PD from nondegenerative parkinsonian syndrome [297]. It is worth noting that, unlike DAT imaging, NM-MRI imaging seems to be also effective for measuring disease progression demonstrating to closely correlate with clinical scores, including UPDRS-III, in advanced PD patients [105,298].

Quantification of QSM (Quantitative Susceptibility Mapping) values in nigrosomes on 3-T scans is likewise effective in the diagnosis of early-stage PD, showing high diagnostic performance in a recent imaging study in 39 patients vs. 25 controls (area under the curve, 0.73) [299].

Nevertheless, although all these MRI techniques provide suitable markers for the nigral structure, they generally may not differentiate PD and other types of degenerative parkinsonism [116]. Further recent neuroimaging techniques, such as diffusion-tensor imaging and density imaging, might help clinicians to differentiate between these conditions but their unavailability on most scanners and the lack of normative databases challenge their use in clinical practice [300,301].

## 11. Conclusions

Imaging of DAT binding is useful in early diagnosis for PD and differentiation from other non-degenerative parkinsonian disorders, but it does not differentiate PD from other degenerative parkinsonisms characterized by dopaminergic cell loss. However, it should be noted that dopaminergic imaging cannot replace the benefits of a careful clinical evaluation. DAT deficit is able to identify the risk of PD onset in patients who have prodromal features such as hyposmia, iRBD or carrying a pathogenic mutation of PD. In patients with PD there is generally a good correlation of DAT levels with bradykinesia and rigidity, and less with tremor, but this seems to be limited to the baseline scans because longitudinal evidence suggests that there is no correlation between change in the striatal DAT uptake and the change in UPDRS motor score. DAT imaging may have some prognostic value, especially in the prediction of motor complications and cognitive dysfunction, but this has limited relevance to current clinical practice. Although DAT-SPECT imaging has been suggested as an enrichment biomarker to be used in early PD neuroprotection trials, it should be noted that some patients with normal DAT binding but motor scores worsening over the years are at risk to be excluded. Finally, clinicians should be aware of issues related to the interpretation of DAT imaging results in view of recent studies questioning the actual relationship between DAT levels and nigral cell or axon counts.

## Figures and Tables

**Table 1:** DAT imaging as a predictive biomarker in PD: motor and non-motor outcomes.

Outcomes	Authors and Year	Type of Study	Radioligand	Population	Main Results
Motor Severity					
UPDRS motor score, Hoen and Yahr scale, Freezing of gait, Falling	Vogt et al., 2011 [224]	Prospective	[^123^I]-β-CIT SPECT	44 early-stage PD	Weak correlation between baseline DAT binding ratios and staging parameters (UPDRS II-III and H&Y) at follow-up
	Ravina et al., 2012 [43]	Prospective	[^123^I]-β-CIT SPECT	491 de novo PD	Lower baseline DAT binding is associated with higher risk for motor-related disability, falling and postural instability
	Rahmim et al., 2016 [227]	Prospective	[^123^I]-FP-CIT SPECT	64 de novo PD	Baseline DAT binding is significantly correlated with UPDRS III and disease duration
	Djaldetti et al., 2018 [222]	Retrospective	[^123^I]-FP-CIT SPECT	41 early-stage PD	Baseline DAT binding does not predict H&Y or UPDRS motor score progression, but lower DAT uptake at baseline is associated with the development of FOG
	Kim et al., 2018 [219]	Retrospective	[^123^I]-FP-CIT SPECT	390 de novo PD	Lower baseline DAT binding in the caudate nucleus and putamen significantly predicts the development of FOG
	Simuni et al., 2018 [70]	Prospective	[^123^I]-FP-CIT SPECT	358 early-stage PD	No correlation between the rate of change of UPDRS-III and change in the striatal DAT uptake
	Li et al., 2018 [184]	Prospective	^11^C-PE2I PET	33 mild-moderate stage PD	Negative correlation between baseline DAT binding in the caudate, anterior and posterior putamen, and motor severity (UPDRS-III, bradykinesia-rigidity subscores)
	Kim et al., 2019 [219]	Prospective	[^123^I]-FP-CIT SPECT	392 early-stage PD	Baseline caudate DAT binding significantly predicts FOG
	Pagano et al., 2019 [230]	Prospective	^11^C-PE2I PET	17 de novo PD, 15 early-stage LD-treated PD, 24 moderate-advanced LD-treated PD	Negative correlation between change in motor severity (UPDRS-III and bradykinesia/rigidity subscores) and change in the baseline DAT uptake in the caudate and posterior putamen
Survival	Makinen et al., 2017 [228]	Retrospective	[^123^I]-FP-CIT SPECT	162 PD: all stages	Baseline DAT binding does not predict mortality
**Motor complications**					
Levodopa-induced dyskinesia (LID) Motor fluctuations	Hong et al., 2014 [87]	Prospective	^18^F-FP-CIT PET	127 de novo PD	Baseline putaminal DAT uptake significantly predicts the development of LID
	Palermo et al. 2020 [88]	Retrospective	[^123^I]-FP-CIT SPECT	105 de novo PD	Lower baseline DAT binding in early-onset PD predicts later motor complications
	Yoo et al., 2017 [233]	Retrospective	^18^F-FP-CIT PET	65 de novo PD	Lower DAT binding in the posterior putamen at baseline is a major risk factor for earlier onset of LID
	Jeong et al., 2018 [234]	Prospective	[^123^I]-FP-CIT SPECT	290 de novo PD	Baseline DAT binding is lower in PD patients who will develop LID
	Chung et al., 2018 [235]	Retrospective	^18^F-FP-CIT PET	342 de novo PD	Lower baseline DAT binding in the anterior putamen predicts the wearing-off phenomenon
	Djaldetti et al., 2018 [222]	Retrospective	[^123^I]-FP-CIT SPECT	41 early-stage PD	Baseline DAT binding does not predict motor complications
	Roussakis et al., 2019 [236]	Prospective	[^123^I]-FP-CIT SPECT	42 de novo PD	Baseline DAT binding does not predict LID
	Chung et al., 2020 [237]	Retrospective	^18^F-FP-CIT PET	205 early-stage PD	Baseline DAT binding, particularly in the anterior putamen, predicts the wearing-off phenomenon
**Non-motor symptoms**					
Anxiety, Depression, Impulsivity, Constipation, Autonomic symptoms, Visual hallucinations, Body weight gain/loss	Ravina et al., 2012 [43]	Prospective	[^123^I]-β-CIT SPECT	491 de novo PD	Lower striatal DAT binding at baseline is associated with depressive symptoms
	Kyoungjune et al., 2019 [238]	Prospective	[^123^I]-FP-CIT SPECT	163 de novo PD	Lower baseline putamen-to-caudate DAT binding ratio is associated with future weight gain in men (but not women)
	La Torre et al., 2020 [239]	Prospective	[^123^I]-FP-CIT SPECT	20 de novo PD	Higher baseline putamen-to-caudate DAT binding ratios are associated with higher emotional responsiveness and higher non-planning impulsivity
	Hinkle et al., 2018 [240]	Prospective	[^123^I]-FP-CIT SPECT	397 de novo PD	Constipation is closely associated with lower baseline caudate DAT binding
	Jaakkola et al., 2017 [241]	Prospective	[^123^I]-FP-CIT SPECT	70 early-stage PD	Baseline low ventral striatal DAT binding is associated with visual hallucinations
**Cognitive performances**					
	Ravina et al., 2012 [43]	Prospective	[^123^I]-β-CIT SPECT	491 de novo PD	Lower striatal DAT binding at baseline is associated with cognitive impairment
	Arnaldi et al., 2017 [242]	Prospective	[^123^I]-FP-CIT SPECT	54 de novo PD	Caudate DAT binding predicts patients’ cognitive worsening at the follow up
	Yousaf et al., 2019 [68]	Prospective	[^123^I]-FP-CIT SPECT	262 de novo PD	Lower caudate DAT binding at baseline is associated with higher risk of cognitive impairment

Retrospective and prospective longitudinal studies evaluating the putative predictive role of baseline striatal DAT binding in PD. Abbreviations: DAT, dopamine active transporter; UPDRS, Unified Parkinson’s Disease Rating Scale; PD, Parkinson’s disease; H&Y, Hoen and Yahr; FOG, freezing of gait; LD, Levodopa; LID, Levodopa-induced dyskinesia; MCI, Mild cognitive impairment.
